# Communities against cancer: a qualitative study assessing the effectiveness of a community engagement initiative in improving cancer awareness for marginalised communities

**DOI:** 10.1186/s12889-025-23179-0

**Published:** 2025-05-31

**Authors:** David Wright, Rebecca Foster, Phoebe Miles, Nicola Duffield, Sally Rickard, Jane Frankland, Lynn Calman, Claire Foster

**Affiliations:** 1 The Centre for Psychosocial Research in Cancer, School of Health Sciences, Faculty of Environmental and Life Sciences, University of Southampton, University Road, Building 67/4061, Southampton, Hampshire SO17 1BJ UK; 2 Wessex Cancer Alliance, Oakley Road, Southampton, Hampshire SO16 4GX UK

**Keywords:** Equality and diversity, Marginalised communities, Cancer awareness, Early detection, Cancer prevention

## Abstract

**Background:**

Marginalised communities (including minoritised ethnic groups and people with lower socioeconomic status) often present with a late cancer stage at diagnosis, affecting survival. This is due to many factors including cultural barriers, mistrust of health services and low levels of cancer awareness. Communities Against Cancer (CAC) aimed to promote cancer awareness and healthy lifestyles and help-seeking behaviours for marginalised communities through a grant-scheme that provided funding for community-led projects, which ran from 1st January 2021 – 31st December 2022. This paper reports findings from a study that assessed whether CAC met its aims and the characteristics of successfully delivered projects.

**Methods:**

A qualitative approach was used involving interviews and observations of meetings and community activities, supported by documentary analysis of minutes, grant applications, reports and quantitative descriptions of grant-level data. Participants included representatives from the funder and grant distributor, community advocates, applicants and recipients of CAC funding. Thirty-seven people were interviewed, all of whom were invited to a second follow-up interview. Twenty-one participants agreed to a second interview, resulting in 58 interviews in total. Interview transcripts and observation fieldnotes were analysed thematically.

**Results:**

CAC community grants encouraged: 1) healthy behaviours (e.g. families on low incomes reported healthier diets); 2) screening and PSA testing uptake (e.g. a group representing neurodivergent people created a film of a breast screening unit, encouraging attendance); 3) awareness of signs and symptoms (e.g. one radio station for South Asian communities broadcast an episode on signs of prostate cancer, their most downloaded programme); 4) help seeking behaviours (e.g. one South Asian community group held meetings with pharmacists, primary care and hospital staff, building trust with local services). Seven characteristics were identified for successful projects (defined as meeting at least one of the initiative’s aims: raising awareness of healthy behaviours, screening uptake, awareness of signs and symptoms, and help-seeking behaviour). The characteristics were: 1) projects are designed with an understanding of the community; 2) effective planning before delivery; 3) projects are co-created with the community; 4) alignment with group values; 5) building community members’ confidence; 6) effective communication; 7) adaptability and evaluation.

**Conclusions:**

The community-based grant model adopted by CAC enabled community members to self-define effective strategies to deliver cancer messages to their communities. As a result, the CAC initiative met its aims of promoting cancer awareness, encouraging healthy lifestyles and help-seeking behaviours by ensuring activites were fully tailored and co-created with marginalised communities.

**Supplementary Information:**

The online version contains supplementary material available at 10.1186/s12889-025-23179-0.

## Background

Early detection and early stage at diagnosis are important factors in cancer survival. Evidence shows that people from marginalised communities often present at diagnosis with a late cancer stage [1]. In England, certain ethnicities (e.g. Caribbean, African or Asian British) have increased odds of late-stage disease at diagnosis compared with White British cohorts for some cancers (e.g. breast, ovarian cancer or colon) [2]. Many factors contribute to this, including cultural and linguistic barriers to accessing healthcare [3], a mistrust of health services due to historical and contemporary discrimination [4], and lower levels of cancer knowledge and awareness [5–7]. A recent systematic review exploring barriers to breast and cervical cancer screening among Black, Asian and minority ethnic women identified numerous factors affecting service uptake, including health service delivery (e.g. health professionals lacking cultural competence), cultural, religious and language issues (e.g. cancer stigma), limited knowledge and awareness (e.g. lack of knowledge of screening services), and emotional factors (e.g. embarrassment and fear) [8].

People with lower socioeconomic status are more likely to present with advanced disease than those with higher socioeconomic status, due to poor symptom knowledge, emotional barriers and de-prioritising medical help-seeking against meeting daily needs [9–11]. Those living with autism and learning disabilities are often marginalised in accessing health services as they can find it difficult to follow health information and communicate needs to healthcare providers [12].

Tailored interventions have been recommended to encourage behaviour change and support cancer screening among marginalised communities [13–15]. These have often adopted educational approaches, such as the US ‘Women Be Healthy’ and ‘Women Be Healthy 2’ eight-week curriculum for women with intellectual disabilities, which increased screening-related knowledge [16]. In a UK study, men with intellectual difficulties were randomly allocated to teaching or information leaflet groups, with the teaching group receiving an educative intervention [17]. Participants were recruited through colleges, day centres, voluntary organisations and youth clubs and the study found that both groups demonstrated improvements in knowledge and skills scores compared with baseline [17].

One US study compared the effectiveness of two modes of self-sampling for cervical cancer screening for women from ethnic minorities: a 30-min visit by a community health worker who demonstrated how to self-sample, and a mailed self-sampling kit with instructions and a pre-addressed, stamped envelope for returning to the laboratory [18]. Both approaches were successful with over 70% screening sample completion rates [18].

Community-based grant schemes have been effective in fostering greater community-led, community-driven activities to increase cancer knowledge and support cancer prevention [19, 20]. However, while these studies have contributed to an understanding of how cancer knowledge and healthy behaviours can be improved, evidence for certain marginalised communities remains limited. A recent Cochrane review of interventions to increase cancer screening for those with severe mental illness found no relevant studies [21]. More research is needed for LGBTQ + communities, the vulnerably housed and refugees, who may face specific barriers to cancer-related knowledge and screening [22].

The Wessex Cancer Alliance Communities Against Cancer (CAC) initiative aimed to promote cancer awareness, healthy lifestyles and help-seeking behaviours for diverse marginalised communities through a community-led grant-scheme. This paper reports findings from a study that assessed the extent to which CAC-funded projects met these aims and the characteristics of those projects that were able to deliver most effectively.

### The communities against cancer intervention

**Fig. 1:**
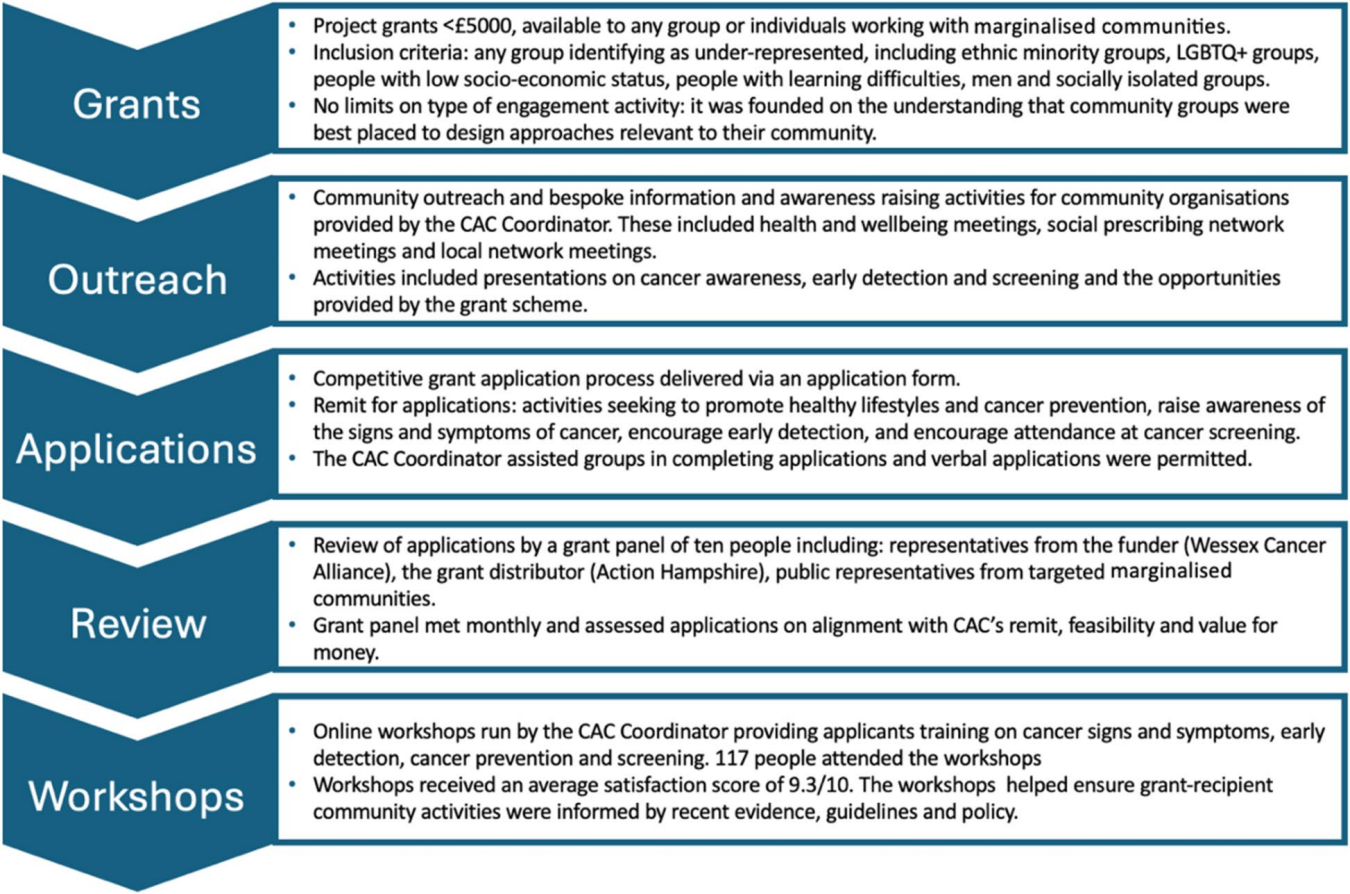
Description of CAC intervention components and process

Communities Against Cancer (CAC) was a community-led grant-delivery initiative that invited applications from individuals and groups from marginalised communities for projects that aimed to promote healthy lifestyles and cancer prevention, increase awareness of signs and symptoms of cancer, and encourage cancer screening and early detection. Funding for CAC grants was provided by Wessex Cancer Alliance, which was funded by NHS England. The initiative purposely had a broad inclusion criteria for applicants, being open to any group that had worse than average cancer-related health outcomes. Applications were open to any charity, community group or social enterprise working in the South of England region. The initiative was launched on 1 st January 2019. Our study assessed the effectiveness of the second round of CAC, which ran from 1 st January 2021 to 31 st December 2022. Figure 1 summarises the components of the intervention. (A CAC Logic Model describing the intervention and intended outcomes is shown in Additional file 1.)

Grant activities were supported by a community-based organisation with established links to diverse communities, comprising a Project Coordinator, a Grant Administrator and a communications and finance team. The total staff resource equated to one full time post. The Coordinator raised awareness of CAC through attending meetings and community events and provided cancer prevention and awareness training. They supported people in the grant application process and grant-holders in their delivery of funded activities. They also worked closely with community advocates (volunteers with an interest in the project, including previous award holders), and local representatives through whom grant information was disseminated.

**Fig. 2:**
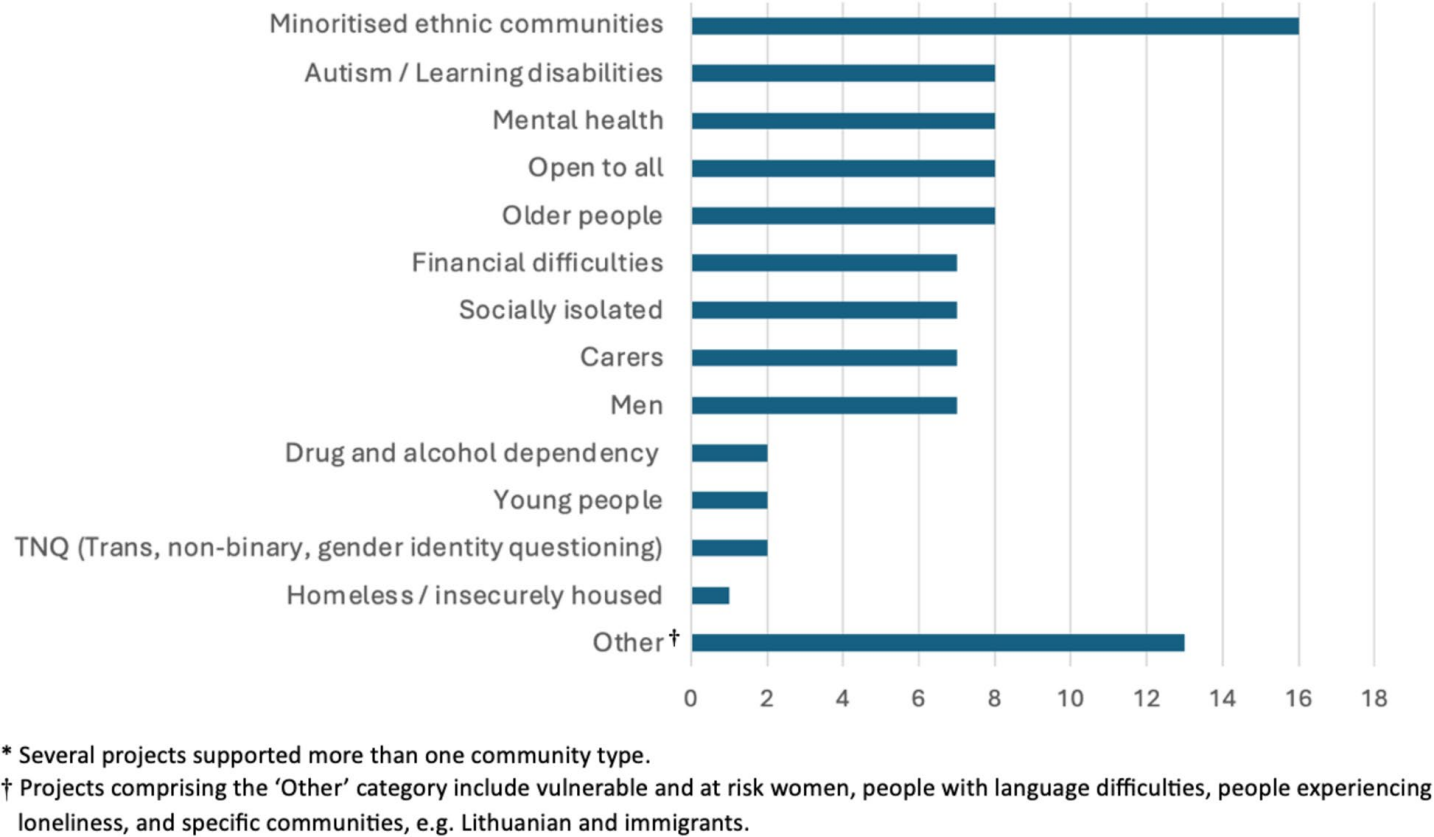
Number of CAC funded projects by community type, 2021–2022*

**Fig. 3:**
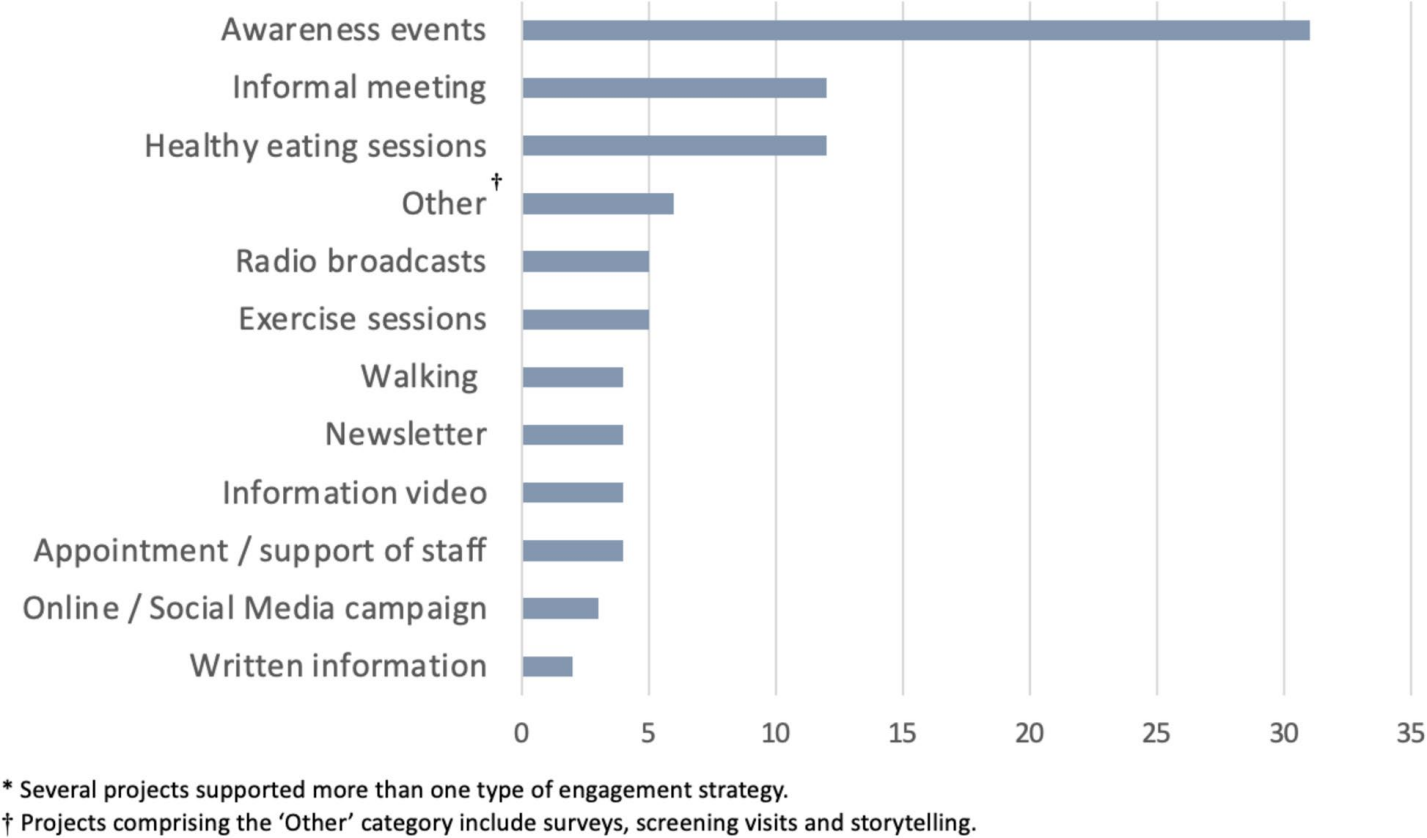
Number of CAC funded projects by type of engagement activity, 2021–2022*

CAC was promoted through social media and websites, sending information fliers to community groups and leaders known to the organisation managing the grant scheme, and attendance at local networking and group events, including religious festivals and open days. A total of 58 applications were considered by the grant panel in the period 1 st January 2021–31 st December 2022 of which 53 were funded, resulting in a success rate of 91.4%. The grant panel consisted of eight members, including advocates for target communities, a representative from Wessex Cancer Alliance, and specialists in public and community involvement. The total value of the 53 successful projects was £206,383. The average size of project funding was £3,894 (range: £760 – £5,400). Ethnic minoritised community groups were the largest community type supported by CAC (16 groups, 30.2%) (Fig. 2). The community engagement strategy most used by grant applicants was awareness events (e.g. courses, workshops, festivals or all-day events), which were used in 31 (58.5%) funded projects (Fig. 3).

### Study aims

The aim of the study was to assess whether the CAC-funded community engagement activity had resulted in a successful outcome, defined in terms of whether it delivered activities to meet at least one of the initiative’s aims:

- Increased healthy behaviours
- Increased screening uptake
- Increased awareness of signs and symptoms of cancer
- Increased help-seeking behaviour

In addition, we assessed the context-specific factors that contributed to the outcomes of successful projects.

## Methods

### Data collection

We used a qualitative approach [23] involving: in-depth qualitative interviews; observations of training sessions, meetings, discussions, funded community activities and other relevant events; and documentary analysis. This was supported by quantitative descriptions of grant-level data.

### Documentary analysis

Documentary analysis was undertaken on all written material relating to CAC. This involved assessing the grant-scheme promotional material and grant-panel meeting minutes. In addition, all applications received were assessed, as were all interim and final reports of funded projects and any grant-level monitoring and evaluation activity. This facilitated the assessment of whether the funded project had met the stated aims of the original application and allowed grant-level evaluation activity to be considered alongside qualitative data. Furthermore, all final reports included a consideration of lessons learned, which supported the assessment of the characteristics of successful grant-holders.

### Interviews and observations

#### Participant selection

We obtained a maximally varied range of participants, including representatives from the following groups:

- The funding organisation and grant distributor
- Community advocates who supported the initiative
- Successful and unsuccessful grant applicants
- Members of community groups in receipt of CAC funding

Thirty-seven people were interviewed, all of whom were invited to a second follow-up interview. Twenty-one participants agreed to a second interview, resulting in 58 interviews in total. Interviews were conducted over 24 months, using an interview schedule informed by the documentary analysis, CAC Logic Model and a review of the literature (Additional file 2). Interviews focused on activites and outcomes that occurred over the period of the grant scheme (1 st January 2021–31 st December 2022). We observed five community events, three training courses for grant applicants and one grant panel meeting. Informed consent was obtained in advance of the first interview and interviews were recorded and transcribed to ensure accurate recall. Transcripts and subsequent reporting were anonymised using the following convention: Participant No./Participant Type (S = Staff; G = Grant applicant or recipient; A = Advocate)/Interview No. (Hence ‘CAC2S 1’ is the first interview with participant number 2, a staff member). ​Most interviews were conducted face-to-face or via Microsoft Teams. RF and DW conducted the interviews, both of whom are experienced qualitative health researchers, independent of CAC and the funding and grant management organisations.

#### Data analysis

We used a theory-driven analysis plan, drawing on the following:

- The CAC Logic Model (Additional File 1). The model informed our analysis by defining the areas of expected outcomes for the CAC intervention and whether these were achieved.
- Positive deviance [24]. Given that certain projects received similar funding and targetted similar communities in the same geographical region, our analysis sought to characterise the projects, understanding the factors that contributed to why some were more successful than others.
- The Consolidated Framework for Implementation Research (CFIR) [25]. This framework was applied to assess differences in outcomes of funded projects and to identify the factors that could account for these.

We analysed interview transcripts and observation field notes thematically, extracting key themes and supporting data. Analysis was initially conducted independently and then collectively by DW and RF to discuss emerging themes, which were reviewed as fieldwork progressed and at the end of data collection.

## Results

Thematic analysis focused on two domains: 1) the extent to which projects delivered against CAC aims (informed by the Logic Model), 2) the characteristics of successful projects (informed by positive deviance and CFIR).

### Delivery against CAC aims

Table 1 provides a summary of the different activities and outcomes from selected projects by core CAC theme areas.

**Table 1:**
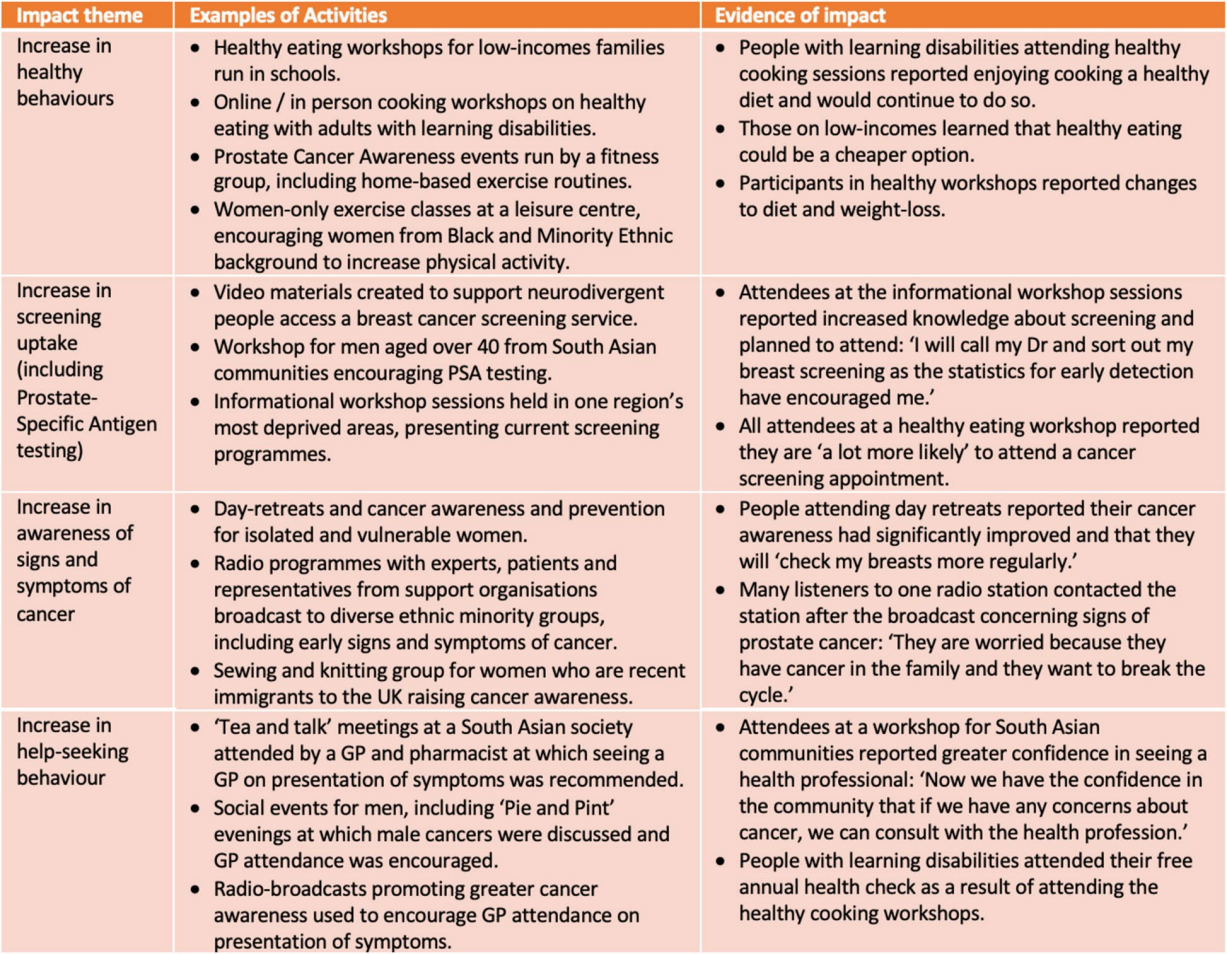
Summary of activities and outcomes of selected CAC projects by intervention aim

#### Activities to promote healthy behaviours

Projects delivered a range of activities to promote healthy behaviours, including encouraging increased physical activity through home-based exercise, promoting Prostate-Specific Antigen (PSA) testing through prostate cancer awareness events, and advocating sun protection through providing free sunscreen samples and information about skin cancer. Twelve projects focused on cancer prevention and healthy diet, involving diverse communities including people with learning disabilities and families on low incomes. Projects typically introduced attendees to new ingredients, built confidence in cooking meals and used workshops as ‘teachable moments’ to explain the link between poor diet and cancer. One community leader working with people with a learning disability commented:

*‘We cooked up healthy alternatives to common things… Lots of our members attended that, it was a really good session, … really well received, they were very enthused about it and open to trying different things.’* (CAC25 R1)

An important message for families on low incomes was that eating healthily was often cheaper than their typical diet, which often consisted of processed foods. One participant commented that the barriers *‘were mainly financial, so learning that the recipes we were making were financially viable and in most cases cheaper and the kids enjoyed them [was important].’* (CAC14 R2).

#### Activities to promote screening uptake and PSA testing

Groups used various strategies to share information on screening and PSA testing and to encourage uptake. One group representing neurodivergent people visited their local hospital breast screening unit and spoke to staff members about what screening involved. This was filmed, edited and shared with group members on WhatsApp, improving members' understanding of the screening process. Another group ran an advert in their community magazine (printed and online), encouraging men from minoritised ethnic communities to get a PSA test. As the director of the group reflected:

*‘I was at a church… and this guy sought me out, he said: “based on your campaign, I had my PSA done.” He said “it’s the most nerve-racking week I’ve ever had… but it came back normal”.’* (CAC12 R2)

Four projects sought to increase awareness and uptake of PSA testing in minoritised ethnic communities. These were particularly effective when supported by health professionals and were linked to local testing services and opportunities.

#### Increase in awareness of signs and symptoms of cancer

Many strategies were used by grant holders to raise awareness of signs and symptoms, including broadcasts by community radio stations, newsletters to members, and using healthy eating workshops as a teachable moment. Radio stations were particularly effective in reaching a large audience for minimal investment. Two radio stations with a combined grant funding of £8,782 reached an estimated audience of over 26,000 listeners, using estimates of listeners supplied by the stations. One radio station director explained:

*‘On the hour, once every four hours, you’ll get a specific message about cancer going out. It might be advice about a specific issue or symptoms, or it might be a particular event that’s coming up* (CAC11 R1)

For one station, their broadcast on signs of prostate cancer became the most downloaded programme with numerous calls to the presenter from concerned family members about symptoms and early detection.

#### Activities to promote help seeking behaviour

CAC grant holders reflected on the need for targeted engagement with communities to encourage help seeking behaviour. One commented on the fear that exists within minoritised ethnic communities: *‘some families, they don’t want to even say cancer from their mouths, they don’t want to say this word and the reason is they are so fearful, they don’t want to be involved with anything cancer related’* (CAC22).

Several projects sought to enhance confidence in seeking help by building trust with local health services. Consultants, doctors and pharmacists took part in radio broadcasts, informal group meetings and information events. As one grant-holder reported, *‘there is definitely a growth in confidence… We have broken the taboo. We are more able to talk to the community’s leaders, GPs, pharmacists about cancer and what the treatments are’* (CAC28 R2). Another project, involving people with learning difficulties, similarly reflected on the importance of establishing effective links with local primary care services, enabling community members to *‘pluck up the courage to book their annual health check’* (CAC25 R1).

### Characteristics of successful projects

Analysis of the funded CAC project identified seven characteristics of highly successful community projects:

#### Projects are designed with a detailed understanding of the community they seek to support

Projects that were designed with an understanding of the target community were more likely to be trusted by group members and be relevant to them, and were thus more likely to be well received. Projects struggled where there was less understanding, e.g. where people were brought in from outside the community group to manage the project. As one participant reflected:

*‘Things haven’t quite worked out as I hoped they would, partly because I’m not familiar with [the group]… it’s trying to work out what I can achieve…, bearing in mind that there are over fifty volunteers involved.’ (CAC6R 2)*

#### Projects are well-planned in advance of delivery

Cancer messages were more likely to be received effectively by group members if the projects were planned effectively in advance of delivery. For many applicants, this involved researching and liaising with experts to develop an engagement strategy, resources and materials: ‘*I had to do quite a lot of digging to find resources specifically for people with learning disabilities… We shouldn’t necessarily just expect them to be out there, but actually they weren’t easy to come by.’* (CAC25R 1). It was evident that those with a considered project design were more likely to succeed.

#### Projects are co-created with trusted community members or partners

Projects were more likely to be better received if they were co-created with community members or partners who were seen to be trustworthy, reputable and credible. This was particularly important for newly-established groups where collaborations with well-known and trusted representatives helped build trust and credibility. One successful project, creating digital resources on cancer screening for people with learning disabilities or neurodiversity, ensured all stages of the project were co-created:

*‘our film producers are all disabled or autistic so they’re bringing the lived experience into it in terms of the areas that might raise anxiety for someone going along to breast screening.’* (CAC3 A 1)

#### Aligning projects with established community group values and activities

Projects were more likely to succeed if they were aligned to the interests of the community group. Community group leaders of successful projects often saw the cancer-messaging as part of a wider programme of activity and engagement and not something to be delivered as a ‘bolt-on’, within the confines of the grant period. Successful projects were also more likely to integrate well into existing processes, routines and formats of community activities. For example, a number of CAC-related activities were timed to coincide with weekly radio shows, regular meetings and annual festivals, thus capitalising on structures already familiar to community members.

#### Building community members’ confidence

Many successful CAC funded projects specifically aimed to build members’ confidence in adopting healthy behaviours and seeking help if needed. This was important for members of some communities for whom cultural, emotional, religious, financial and other barriers can undermine confidence in changing behaviours. The healthy eating projects, for example, built confidence in menu planning and preparing and eating healthy ingredients. Other projects aimed to build confidence in accessing health services, such as creating online videos of a breast screening unit for neurodivergent people.

Several interviewees reflected that it was not sufficient to give people information: they had to address the underlying concerns of community members. They needed to *‘break the taboo,’* and give people confidence to discuss cancer: *‘if there is somebody of their community talking, then they feel a little bit more reassured, and they can ask more questions’* (CAC28S 1). Involving health professionals in awareness and engagement activities, such as local GPs and consultants, was particularly effective in engendering trust.

#### Effective communication with group members and external partners

Most groups had effective internal communications and excellent networks of external partners, including local charities and health providers. This ensured that stakeholders knew about CAC activities and external presenters were able to tailor discussions to group needs. Successful project leaders were aware of the need to frame the cancer messaging in ways that would be well-received by group members. Healthy eating workshops, for example, were an effective mechanism for communicating cancer messages to people with a learning disability as these could be delivered in an engaging way alongside practical activities. One member of a South Asian community group discussed the importance of supporting people in discussing cancer messages in ways that were meaningful for them, for ‘*they can ask more questions, and they’ll be explaining [in] the way that they would understand.’ (CAC28S 1).*

#### Adaptability and evaluation

It is inevitable that unforeseen challenges occur when delivering projects, particularly given that many activities occurred during the rapidly shifting restrictions of COVID. Projects that were adaptable to the changing demands of delivery were typically successful in meeting project aims. For example, one project delivering healthy eating cookery classes for people with learning disabilities successfully adapted the session to online delivery during the COVID pandemic.

A related, important characteristic of successful projects was the ability to pilot, test, reflect and evaluate community engagement activities. Part of effective project design was defining with clarity the success measures for funded activity and then assessing the extent to which those measures had been met. If CAC activities were not delivering as intended, an important skill was to reflect and try a different approach. Several grant-holders were experienced in monitoring and evaluating their own project, for example devising their own surveys to capture the degree to which CAC aims were met. For those less experienced, advice on monitoring and evaluation techniques was given by the Project Coordinator through online training.

## Discussion

Improving early diagnosis of cancer is a high strategic priority globally. The drive for early detection is particularly important for marginalised communities where cultural barriers, discrimination and limited knowledge mean members of these communities are more likely to be diagnosed with cancer at a later stage than the general population [10].

Research into increasing cancer awareness, building confidence and encouraging healthy behaviours with marginalised communities is developing rapidly. Our study contributes to this body of knowledge by assessing the effectiveness of a community-based initiative to deliver cancer awareness information and activites to community groups. CAC reflects the recommendations from research by supporting engagement activities that are tailored to the needs of marginalised communities [13]. The CAC grant scheme allowed representatives from under-served communities to apply for funds to deliver cancer messages in ways that were most relevant to their members. The qualitative assessment of the scheme’s effectiveness underscores the importance of community-led and community-driven initiatives in delivering cancer awareness, and health-seeking information and activities in marginalised communities. The grant scheme was able to meet its aims as it was founded on the principles of co-creating engagement activities with marginalised communities. These principles are the hallmark of community-based participatory research and are effective in reducing cancer health disparities [26]. Through such community-based participation, the grant scheme was able to build the awareness of healthy behaviours and signs and symptoms of cancer, and the importance of help-seeking behaviour.

Typical engagement strategies for CAC-funded projects were educational in focus, including online and in-person events, meetings, programmes and workshops, which reflects the published literature where educational interventions were commonly used [16, 17]. The literature also reveals that evidence for certain marginalised communities is very limited, including LGBTQ + people, neurodivergent people and the vulnerably housed [22]. In this regard, it is notable that the community-based grant-funding approach adopted by CAC was successful in reaching these communities. However, while applications from minoritised ethnic communities were well represented, other communities including lesbians and gay people and those vulnerably housed were less well represented.

Applying the principles of positive deviance [24] and the Consolidated Framework for Implementation Research [25] to the analysis enabled the characteristics of highly successful projects to be identified, including aligning with community group values and being adaptable to change. These characteristics reflect wider literature on sustaining effective community engagement in the design and delivery of health services [27] and in public health research [28]. This literature similarly details the importance of early and committed engagement, shared decision-making, building confidence of community members and ensuring clear and effective communications [27, 28].^.^Furthermore, it is striking how closely the characteristics of highly-successful CAC projects reflect those identified in the quality improvement literature, where clearly stated aims, aligned vision, appropriateness of design, effective communication, effective evaluation and adaptability to delivery in practice are important qualities when seeking to change practice [29–31].

The CAC grant-funding approach gave autonomy for the projects to define for themselves the most appropriate ways to engage with their communities, thus delivering a tailored approach to community engagement, as recommended in the literature [13–15]. This tailored approach helped to ensure engagement with community members was effective, thus delivering cancer messages in ways that were aligned to community needs and experience. As a result, CAC-funded projects were successful in delivering activities that raised awareness of the signs and symptoms of cancer and promoting healthy lifestyles and help-seeking behaviours.

The CAC scheme is a sustainable model and, indeed, further rounds of grant-funding have been allocated on the basis of study findings presented here. Furthermore, grant-holders from earlier rounds have successfully applied for further CAC funding, enabling them to extend the reach and impact of their activities. In addition, the CAC grant scheme is replicable. Given the successful delivery of information and awareness through CAC funding relating to cancer awareness, healthy behaviour and health seeking activities, the grant-scheme model warrants consideration for adoption in other regions and for other health conditions (e.g. heart disease).

### Limitations

While the qualitative approach undertaken was effective in eliciting the experience and effectiveness of community engagement activities, several limitations should be noted. The approach was appropriate in identifying the short- to medium-term outputs from CAC-funded activities. However, the longer-term outcomes from these activities, such as the influence on screening attendance, were beyond the timeframe of this study. While it was possible to explore the influence of certain projects on attendees (e.g. workshops), it was not possible with the study design to capture responses for other recipients of cancer messages (e.g. listeners to radio stations). Furthermore, the findings from the study would have been strengthened further by interviewing more participants in CAC-funded activities relative to grant recipients. In addition, the ‘intention-behaviour gap’, widely recognised in health literature, acknowledges that intended actions may not be realised in actual behaviour [32]. Finally, there is a risk of bias in gathering qualitative information from grant recipients. This was addressed by exploring challenges by a team of experienced researchers and the assurance of confidentiality. No such bias was evident in the analysis.

## Conclusion

Improved rates of early detection of cancer for marginalised communities remains a high priority within the UK and internationally. Community engagement is needed to encourage healthier lifestyles, increase cancer knowledge and improve help-seeking behaviours. An adaptable, tailored approach, co-created with marginalised communities is recommended. The CAC community-based grant scheme adopted such an approach, resulting in the successful delivery of activities and information to support behaviour change. For community grant-holders, a clearly stated plan and aims, aligned vision, effective communication and adaptability to delivery in practice were important factors in assuring successful delivery. These characteristics of successful projects should be considered when funding or delivering community engagement activities for marginalised communities.

## Supplementary Information

Supplementary Material 1.

Supplementary Material 2.

## Data Availability

The data that support the findings of this study are available on request from the corresponding author (D.Wright@soton.ac.uk). The data are not publicly available due to privacy or ethical restrictions. Some interview data will be redacted where they contain information that could compromise participant anonymity.

## Abbreviations

CAC: Communities Against Cancer
CFIR: Consolidated Framework for Implementation Research
LGBTQ +: Lesbian, Gay, Bisexual, Transgender, Queer or Questioning plus
PSA: Prostate-Specific Antigen
TNQ: Transgender, non-binary, gender identity questioning
