# Supplementary figures and images for: Communities against cancer: a qualitative study assessing the effectiveness of a community engagement initiative in improving cancer awareness for marginalised communities
Source: BMC Public Health. 2025 May 31;25:2011. doi: 10.1186/s12889-025-23179-0 (PMC12125787; doi:10.1186/s12889-025-23179-0)

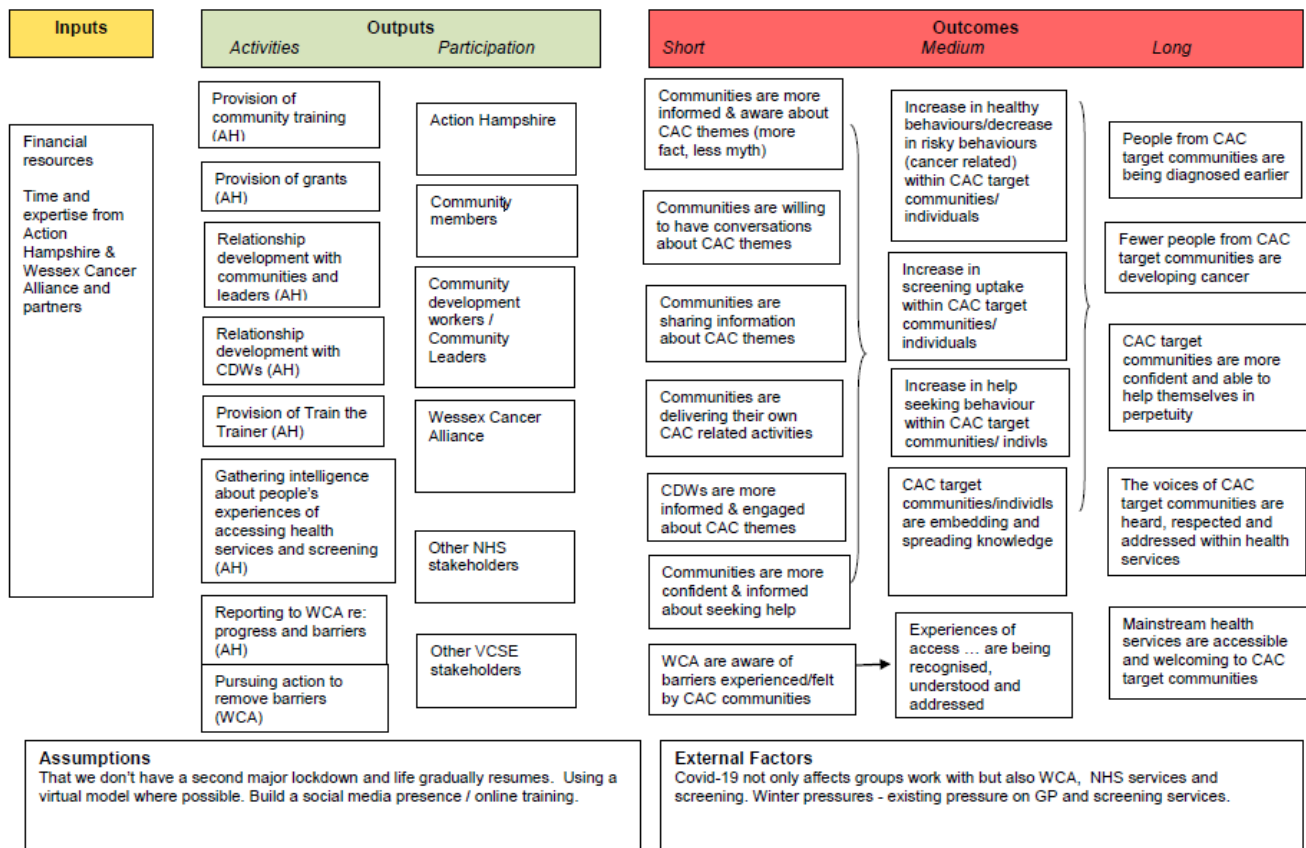

Supplement: Supplementary file 1 — Supplementary Material 1. [file 12889_2025_23179_MOESM1_ESM.pdf]
