# Supplementary material for: Communities against cancer: a qualitative study assessing the effectiveness of a community engagement initiative in improving cancer awareness for marginalised communities
Source: BMC Public Health. 2025 May 31;25:2011. doi: 10.1186/s12889-025-23179-0 (PMC12125787; doi:10.1186/s12889-025-23179-0)
Supplement: Supplementary file 2 — Supplementary Material 2. [file 12889_2025_23179_MOESM2_ESM.pdf]

Communities Against Cancer question schedule – recipients of support

This question schedule is a guide, involving a range of issues to be covered. The exact way questions are asked will be adapted in response to the participant's answers, enabling experiences to be explored in depth.

Prior to interview / focus group

Consent

1. Invitation and information sheet – check the participant has received a copy
2. Read each statement and ask the participant if they agree / disagree
3. Reinforce: 'You can ask to stop at any time'
4. Assure confidentiality
5. Start recorder and check it is recording

Process Evaluation: design and delivery of Communities Against Cancer

I'd like to start by asking you questions about Communities Against Cancer itself and your experiences of the support you have received.

1. How did you hear about Communities Against Cancer? How did you get involved?

2. What do you think it is that Communities Against Cancer is trying to do?

   Prompt
   a. What do you think Communities Against Cancer is trying to achieve?
   b. If the interviewee is not aware of the aims of Communities Against Cancer, the interviewer should remind them the aims of the project are to:
      - promote healthy lifestyles and prevent cancer
      - raise awareness of the signs and symptoms of cancer
      - improve early cancer detection and encourage cancer screening
      - reach people at risk of poor health outcomes because of barriers to accessing health services/healthy lifestyles
   c. Do you think what Communities Against Cancer is trying to do could work in your community? Why / why not?

3. What support have you received from Communities Against Cancer?

   Prompt
   a. Ascertain whether they have received support through the grant scheme, face-to-face / virtual training, building relationships, emails, telephone, virtual meetings, the Facebook group, support from an advocate supported by Communities Against Cancer, e.g. a Community Development Worker, and / or anything else.
   b. If you received a grant, what was this for?

4. [The following questions will be selected according to the type of support received.]

   a. Grants
      - How did you find the process of applying for a grant?
      - What do you think of the grant form you had to complete?
      - Were you given enough information?
      - Did anyone help you with the form?
      - How helpful were they?

   b. Training
      - What do you think of the training you received?
      - Was the content appropriate / sufficient for what you needed to know?
      - Do you remember who provided the training? How helpful were they?
      - Did you have any problems accessing the training (online / physically)?
      - What was the most important thing you learned from the training?

   c. Relationship Building
      - With whom have you built relationships at Communities Against Cancer / Action Hampshire / locally?
      - How helpful have these relationships been?

   d. Communities Against Cancer advocates (e.g. Community Development Workers)
      - Which seldom heard groups do you work with?
      - Have you promoted the Communities Against Cancer project to any groups or individuals you work with? Who?
      - Have you encouraged any groups or individuals that you work with to apply for a Communities Against Cancer grant/ attend a Communities Against Cancer workshop or speak to one of the Communities Against Cancer team? Please explain how.
      - How helpful has the support you have received from Communities Against Cancer / Action Hampshire been? In what way?
      - What additional support do you think you need in promoting the Communities Against Cancer project?

5. If you could make one improvement to the support you have received from the Communities Against Cancer team, what would it be? Are there any other improvements?

Outcome evaluation: what difference is Communities Against Cancer making?

I'd now like to ask questions about whether the support you have received has made any difference to your understanding of cancer.

6. Has [the grant activity / the training / your involvement with Communities Against Cancer / your Community Leader] increased your awareness of the signs and symptoms of cancer?

   Prompt
   a. If yes, can you give an example of how your awareness increased?
   b. If no, why hasn't it increased?
   c. What are main signs and symptoms?
   d. [Where appropriate] Has it increased awareness for your group or community? If so, how? If not, why?

7. Has [the grant activity / the training / your involvement with Communities Against Cancer / your Community Leader] increased your confidence to get advice or help or to go for screening?

   Prompt
   a. If yes, can you give an example of how your confidence has increased?
   b. If no, why hasn't it increased?
   c. [Where appropriate] Has it increased confidence for your group or community? If so, how? If not, why?

8. Have you adopted a more healthy lifestyle as a result of [the grant activity / the training / your involvement with Communities Against Cancer / your Community Leader]?

   Prompt
   a. If yes, how have your healthy behaviours changed (e.g. diet, exercise)? What do you do differently?
   b. If no, why haven't they changed?
   c. Have you stopped doing things that may increase the risk of getting cancer (e.g. smoking, diet)? If yes, what? If no, why?
   d. What are the main ways to reduce the risk of getting cancer?
   e. [Where appropriate] Have members of your group or community adopted a more healthy lifestyle as a result of Communities Against Cancer? If yes, in what way? If not, why?

9. Has the support you have received from [the grant activity / the training / your involvement with Communities Against Cancer / your Community Leader] changed your awareness or attitudes about cancer screening?

   Prompt
   a. If yes, in what way?
   b. If no, why not? Are there continuing barriers? If so, how might they be addressed?
   c. [Where appropriate] Has the support received from Communities Against Cancer changed levels of awareness or attitudes about cancer screening for your group or community? If so, how?

10. Do you know where to go to if you needed help for a health-related problem? Has [the grant activity / the training / involvement with Communities Against Cancer / your Community Leader] provided any knowledge of where to go if you had any health concerns?

    Prompt
    a. Who would you see if you thought you had possible signs of cancer?
    b. How confident are you in seeking help for a health-related problem?
    c. Have you used any health services as a result of [the grant / the training / your involvement with Communities Against Cancer / your Community Leader]? What was your experience of the health service?
    d. [Where appropriate] Do people in your group or community know where to go to get help for health-related problems? Has Communities Against Cancer helped with this? If yes, in what way? Has it resulted in anyone deciding to get help?

11. Have you received any information about cancer, signs and symptoms and prevention through your involvement in Communities Against Cancer?

    Prompt
    a. If yes, what information did you receive?
    b. How useful or helpful was this information?
    c. Was this information shared with anyone else in your group / community?
    d. Was this information shared with anyone else, e.g. work colleagues, neighbours

12. Do you feel you have been properly listened to regarding your experiences of healthcare?

    Prompt
    a. If yes, was that because of your involvement with Communities Against Cancer
    b. If yes, do you feel your feedback will reach the right people? Please explain
    c. If no, please explain why not

13. [For interviewees who are members / leaders of a group] Do other people in your group / community know anything about Communities Against Cancer?

    Prompt
    a. If yes, is there a shared understanding of what Communities Against Cancer is trying to achieve?
    b. If no, what can be done to promote the aims of Communities Against Cancer?

[***Long-term outcomes asked towards the end of the Communities Against Cancer project***]

14. Has your involvement with Communities Against Cancer had any impact on how confident you feel in managing your health?

Prompt

a) Do you feel more confident in managing your health to reduce the risk of getting cancer?
b) Do you feel more confident in managing your own health-related problems?
c) Do you feel more confident in managing your health to stay well and avoid health problems in the future?

15. To your knowledge, has the support provided by [the grant activity / the training / your role as Community Development Worker / your Community Leader] led to anyone being diagnosed with cancer earlier?

Conclusion

16. Is there anything we haven't covered that you would like to add?

Close of interview

- Turn recording equipment off
- Reconfirm consent given
- Thank the participant
- Explain what happens next
- Ask if there are any further questions about the evaluation.

Communities Against Cancer Interview schedule – Staff

This interview schedule is a guide, involving a range of issues to be covered. The exact way questions are asked will be adapted in response to the participant's answers, enabling experiences to be explored in depth.

Prior to interview

Consent

1. Invitation and information sheet – check the participant has received a copy
2. Read each statement and ask the participant if they agree / disagree
3. Reinforce: 'You can ask to stop at any time'
4. Assure confidentiality
5. Start recorder and check it is recording

Process Evaluation: design and delivery of Communities Against Cancer

1. To what extent has it been possible to deliver Communities Against Cancer as originally planned?

   Prompt
   a. Have there been any variations in how Communities Against Cancer has been delivered from the initial plan (e.g. grant scheme, training, building relationships, supporting Communities Against Cancer advocates)? (Probe what changes have been made and why).

2. Can you describe what types of support have been provided to seldom heard groups, specifically relating to:

   a. the grant scheme
   b. face-to-face / virtual training
   c. relationship building (probe here precisely how this happened: what relationships, with whom and how?)
   d. supporting Community Development Workers
   e. Anything else?

3. How well do you think those individuals and groups supported by Communities Against Cancer understand the aims of the initiative?

   Prompts
   a. Is there a shared understanding of the aims of Communities Against Cancer across grant recipients, Communities Against Cancer advocates, individuals and groups supported by the project?
   b. Do those supported by Communities Against Cancer understand why healthy lifestyles, awareness of symptoms, early detection, cancer screening and early presentation of symptoms are important?
   c. Do they understand why it is important to raise awareness of these issues among seldom heard groups?

   d. How informed and engaged do you think the Communities Against Cancer advocates are?

4. Have there been any challenges or barriers to delivering Communities Against Cancer?

   Prompt
   a. What are they?
   b. Were they resolved? If so how?

5. Have there been any enablers that helped the delivery of Communities Against Cancer?

   Prompt
   a. What are they?
   b. Has learning from these enablers been shared with the rest of the team?

6. What are the costs of delivering Communities Against Cancer in terms of: money, time, equipment, people? How do they compare to what was originally planned?

7. Which aspects of the Communities Against Cancer project have worked well (e.g. grant scheme, training, relationship building)? Why?

8. Which aspects of the Communities Against Cancer project have worked less well and requires improvement (e.g. grant scheme, training, relationship building)? Why?

Outcome evaluation: what difference is Communities Against Cancer making?

9. Do you think Communities Against Cancer has increased awareness of the signs and symptoms of cancer among seldom heard groups?

   Prompt
   a. If yes, can you give an example of how awareness has increased?
   b. If no, why hasn't it increased?

10. Do you think that Communities Against Cancer has changed healthy behaviours among seldom heard groups in any way?

   Prompt
   a. If yes, can you give an example of how healthy behaviours have changed?
   b. If no, why haven't they changed?
   c. Are there any examples of changes to risky behaviours among seldom heard groups?

11. Do you think Communities Against Cancer has changed awareness, attitudes or behaviours related to attending cancer screening?

   Prompt
   a. If yes, in what way?

   b. If no, why haven't they changed?

12. Has Communities Against Cancer made any difference to how confident and informed seldom heard groups are in seeking help for health-related problems?

   Prompt
   a. If yes, how and why?
   b. Has it made any different to people's confidence to manage their own health?
   c. Has there been a move to more planned care?

13. To what extent do you think seldom heard groups supported by Communities Against Cancer share information about signs and symptoms, healthy and risky behaviours, seeking help and cancer screening? Why?

[Long-term outcomes asked towards the end of the Communities Against Cancer project]

14. Have cancer health services used intelligence from this project about the experiences and attitudes of seldom heard groups to inform or shape services they provide? If so how?

15. Do you think the Communities Against Cancer approach is the right one for promoting healthy lifestyles, raising awareness, and encouraging help-seeking behaviours?

   Prompt
   a. If yes, why?
   b. If no, what changes would you make to its design?

16. How appropriate and feasible would it be to replicate Communities Against Cancer to other regions / conditions?

   Prompt
   a. What are the enablers / challenges to wider replication?

Conclusion

17. Is there anything we haven't covered that you would like to add?

Close of interview

- Turn recording equipment off
- Reconfirm consent given
- Thank the participant
- Explain what happens next
- Ask if there are any further questions about the evaluation.
